# The impact of the COVID-19 epidemic on mental health of undergraduate students in New Jersey, cross-sectional study

**DOI:** 10.1371/journal.pone.0239696

**Published:** 2020-09-30

**Authors:** Aleksandar Kecojevic, Corey H. Basch, Marianne Sullivan, Nicole K. Davi

**Affiliations:** 1 Department of Public Health, College of Science and Health, William Paterson University, Wayne, NJ, United States of America; 2 Department of Environmental Science, College of Science and Health, William Paterson University, Wayne, NJ, United States of America; Ryerson University, CANADA

## Abstract

**Objective:**

The COVID-19 pandemic has been a period of upheaval for college students. The objective of this study was to assess the factors associated with the increased levels of mental health burden among a sample of undergraduate college students in Northern New Jersey, the region of the U.S. severely impacted by the outbreak of COVID-19.

**Methods:**

College students (N = 162) enrolled in an introductory core curriculum course completed a cross-sectional survey. The survey collected information on demographics, knowledge levels and sources of COVID-19 information, behavior changes, academic and everyday difficulties, and mental health measurements (depression, anxiety, somatization, and stress). Multivariable regression analysis was performed to identify factors associated with mental health outcomes.

**Results:**

Descriptive findings indicate that students have a fundamental knowledge of COVID-19 transmission and common symptoms. Students tend to use and trust the official sources and have changed their behaviors in accordance with public health recommendations (i.e., increased hand washing, wearing mask). However, students reported a number of academic and everyday difficulties and high levels of mental health distress. High levels of depression were associated with difficulties in focusing on academic work and with employment losses, while higher levels of anxiety were more likely to be reported by students other than freshmen and those who spend more than one hour per day looking for information on COVID-19. Inability to focus on academic work and an elevated concern with COVID-19 were more likely to be associated with higher levels of somatization, while trusting news sources was associated with lower levels of somatization. Those with higher levels of perceived stress were more likely to be females, unable to focus on academic work, and report difficulties in obtaining medications and cleaning supplies.

**Conclusions:**

The COVID-19 pandemic is making a significant negative impact on mental health of college students. Proactive efforts to support the mental health and well-being of students are needed.

## Introduction

The novel coronavirus (COVID-19) pandemic has rapidly spread across the globe, causing massive disruptions to everyday life, including in the United States (U.S.). According to the Center for Systems Science and Engineering (CSSE) at Johns Hopkins University [1], as of April 27^th^, there were close to a million of confirmed cases with a total of 55,952 deaths in the U.S., with New Jersey among the hardest hit states in the country reporting 111,188 confirmed cases and 6,044 deaths [2]. At the time of this study, the counties in Northern New Jersey, where this study took place, have some of the highest confirmed case counts in the state.

The pandemic has the potential to affect college students physically, academically, financially and psychologically. Some colleges and universities in New Jersey have reported that a number of students have tested positive for COVID-19 [3–6]. In order to prevent widespread transmission of the COVID-19 virus among staff and the young adult population, higher-education institutions across the country have rapidly switched from in-person to online learning [7]. In a short period of time, college students’ lives have dramatically changed as they have been asked to leave campus, adjust to new living circumstances, and adapt to online learning platforms. The switch to online learning, particularly in courses that were not originally designed for on-line delivery likely has increased stress among students. Courses designed to include high levels of interaction and hands-on experiences such as practicums, labs, and/or artistic performance have a clear disadvantage in regards to the evaluation of students [8]. Some students may have difficulties with access to computers and the internet at home [8, 9]. Additional challenges include concerns about their health, health of family members, and worry about finances, particularly among those who support themselves by working in industries severely impacted by prolonged closures such as retail or the service industry. According to a survey by the loan management website Student Loan Hero [10] 4 out of 5 college students are facing financial difficulties due to the COVID-19 pandemic. Further, most college students are not eligible for immediate financial relief under the Coronavirus Aid, Relief and Economic Security (CARES) Act [11].

College students’ mental health has been rising concern with a significant number of students experiencing psychological distress [12]. Mental health issues can significantly impair students’ academic success and social interactions affecting their future career and personal opportunities. The rapid spread of COVID-19 and social distancing measures imposed across the country are expected to further affect the mental health of the population, including college students. Several studies have examined the psychological impact of the pandemic on the general public [13–15], health care workers [16], or older adults [17]. These surveys revealed greater concerns about social isolation, and increased stress, anxiety, and depression among respondents. Interestingly, a study of Chinese general public [13] also reported that exposure to accurate content of health information during the epidemic was associated with lower stress levels. Conversely, the studies of previous epidemics show that inaccurate information, particularly prevalent in online discussion boards or social media, was associated with anxiety and fear [18]. Accurate information, knowledge and perceptions of risk can contribute to behavioral changes [19], such as social distancing, which is currently one of the few ways to mitigate the spread of COVID-19. The current evidence also shows that taking prescribed precautionary measures (i.e. hand hygiene, mask wearing) to prevent the spread of COVID-19 may reduce negative psychological impacts by providing a sense of security to those engaging in these preventative actions [13]. While many colleges and universities provide students with information related to COVID-19 from reliable sources [20], there is a high potential for misinformation and disinformation to spread through online sources and social media often used by young adults [21–23] further contributing to stress, anxiety and depression among students.

Available literature examining the impact of the COVID-19 epidemic on students in the U.S. is primarily focused on the role of medical and other health profession students during the current COVID-19 epidemic [24–26]. Limited research examining the impact of the COVID-19 epidemic on the psychological health of students has primarily focused on students outside the U.S. Furthermore, little is known about factors contributing to psychological distress related to COVID-19 among college students. One study of undergraduates of Changzhi medical college in China, indicated that a quarter of those surveyed have experienced anxiety due to COVID-19 [27]. Having a relative or an acquaintance infected with COVID-19, economic stressors and academic delays were all positively associated with an increased level of anxiety symptoms. A previous study investigating adverse psychological reactions to the Middle East Respiratory Syndrome Corona Virus (MERS-CoV) outbreak in 2014 among medical students in Saudi Arabia indicated that increased hygienic habits and social distancing were positively associated with the psychological well-being of students [28]. Additionally, a study of Iranian medical students found a high level of COVID-19 related knowledge, engagement in self-reported preventive behaviors, and moderate risk perception among respondents [29].

### Current study

In light of growing concerns related to the impact of COVID-19 on the mental health of vulnerable groups [30], there is an urgent need for research to address mental health burden of the COVID-19 pandemic on college students. At the time of writing we were unable to identify published research on the impact of the COVID-19 pandemic on the mental health of undergraduate college students in the US. Therefore, the current investigation had two primary aims. First, we sought to determine the levels of mental health distress among undergraduate college students during a time period when the significant number of people in Northern New Jersey were testing positive and dying due to COVID-19. Second, we sought to examine whether factors such as knowledge, sources of information, and academic and everyday difficulties are associated with mental health distress among college students during the COVID-19 pandemic.

## Methods

### Sample

This cross-sectional survey was administered at a suburban public university in Northern New Jersey, U.S., in April of 2020. All students (n = 450) taking an introductory core curriculum course focusing on personal health and delivered by the Department of Public Health were invited to participate in this study. While completion of this course is required for admission to a Public Health program, students from other majors may take either this course or other courses focused on personal well-being. The instructors of 17 course sections were provided with a description of the study and were asked to alert their students about the forthcoming invitation. A link to the survey was delivered to students via e-mail, and two reminders were sent in the subsequent week following the initial invitation. Participants provided informed consent to participate in an anonymous survey by completing and submitting the questionnaire electronically in Qualtrics software (Qualtrics, Provo, UT). All data were self-reported. A total of 162 students completed the survey, resulting in a response rate of 36.0%. Students were given an opportunity to enter a raffle to win one of five $20 gift cards. The University Institutional Review Board (IRB) approved the study.

### Measures

#### Demographics

Demographic information included age (recorded as continuous variable), sex (male, female, other), ethnicity (Hispanic/non-Hispanic), race, class level (freshman, sophomore, junior, senior), and whether the student is a health major—public health or nursing (yes/no).

#### Knowledge related to COVID-19

A total of 10 knowledge questions were asked, 7 of which were True/False (e.g., “Those who are elderly and have chronic illnesses are more likely to be severe cases”), while 3 questions assessed the level of agreement with the statements (e.g. “To prevent infection with COVID-19, people should avoid going to crowded places”) on a 5-point scale (0 = “*strongly disagree*” to 4 = “*strongly agree*”). All knowledge questions were based on Centers for Disease Control and Prevention (CDC) fact sheets [31].

#### Sources of information on COVID-19

Students were asked what information sources (i.e. news websites, doctors, friends) they trust to provide accurate COVID-19 information (check all that apply). Additionally, students were asked how often do they use these sources to get health information on COVID-19 on a 5-point scale that ranges from 0 =“*never*” to 4 =“*always*”. Values were dichotomized to 0 (never, rarely, sometimes) and 1 (often, always). In addition, they were asked to indicate the average number of hours per day they spend looking for information on COVID-19.

#### Engagement in precautionary behaviors

Using 5-point scale (0 = “*not at all*” to 4 = “*extremely*”), participants were asked whether they engaged in precautionary changes of behaviors related to their personal hygiene (e.g. “I have increased hand washing”) or social habits (e.g. “I have limited going outside only to essential trips”) since the start of the pandemic. The statements were formed based on previous literature [13]. Values were dichotomized to 0 (did not engage in precautionary behaviors at all) and 1 (engaged in in precautionary behaviors even a little bit).

#### Hardships experienced during the pandemic

Students were asked to indicate whether they experienced various academic (i.e. difficulties with online learning, ability to focus on academic work, etc.), or life (i.e. losing job, getting food, etc.) difficulties. Some values were combined for easiness of presentation (i.e. difficulties with online learning and inadequate WiFi/Internet access).

### Mental health burden

Level of concern with the current pandemic was assessed by a single question asking how concerned the respondent feels about COVID-19. Responses were recorded on a 5-point scale that ranges from 0 =“*not at all*” to 4 =“*extremely*”. Values were dichotomized to 0 (not at all to moderately concerned), and 1 (very to extremely concerned).

To assess levels of depression, anxiety and somatic distress, we used the Brief Symptom Inventory, BSI-18, [32]. The assessment measures self-reported psychological distress in the prior week. Participants were queried about the extent to which something has distressed or bothered them in the past 7 days (e.g., “In the past 7 days, how much were you distressed by feeling lonely?”) on a 5-point scale ranging from 0 =“*not at all*” to 4 =“*extremely*”. As per the BSI-18 scoring instructions, raw scores were converted to T scores using gender specific community norms. Higher scores signify greater levels of depression, anxiety, or distress arising from perceptions of bodily dysfunctions, i.e. somatization. Because the BSI clinical case cutoff likely has a low positive predictive value in this population [33], we are not reporting on prevalence of psychological distress in our sample. Cronbach’s alpha for the overall scale was 0.94 (α = 0.89, 0.89, 0.84 for the depression, anxiety, and somatization subscales, respectively).

The Perceived Stress Scale (PSS), a 10-item self-report questionnaire with strong reliability and validity [34], was used to assess general stress appraisal. Respondents were asked to specify how often they have felt or thought a certain way in the past month (e.g., “In the last month, how often have you been upset because of something that happened unexpectedly?”) on a 5-point scale ranging from 0 =“*never*” to 4 =“*very often*”. Some of the items were scored in reverse. Responses were then summed to indicate the level of perceived stress. It should be noted that the PSS is not a diagnostic instrument and there are no score cut-offs. Nevertheless, higher scores signify a greater perceived stress. Cronbach’s alpha was 0.84.

### Data analysis

Statistical analyses were conducted using the Statistical Package for Social Sciences, SPSS, 26.0 (IBM). Descriptive analyses examined the distribution of all variables of interest. Eleven participants did not complete questions about mental health burden and stress; hence they were not included in bivariate and regression analyses. Using t-tests for means we then examined unadjusted bivariate associations between the independent variables and the four variables describing mental health burden. Next, we entered all bivariate correlates that achieved significance of less than 0.05 into multivariable linear regression models to evaluate the predictive nature of independent variables on depression, anxiety, somatization and stress while controlling for the potentially confounding effects of other variables in models. To ensure that covariates were not collinear, we examined the variance inflation factors for each variable, none of which were greater than two, indicating low levels of collinearity. Standardized β regression coefficient, 95% confidence intervals (CIs), and *p*-values are reported, together with the test-statistic for each of four models.

## Results

### Descriptive findings

As shown in **Table 1**, the median age of our sample was 19 years (range 18–37). Study participants were predominantly female (71.0%) and non-White (63.0%). Two-thirds of the participants were freshman, while approximately one third were health majors.

**Table 1:** Demographic information of respondents (N = 162).

Variable	Category	N (%)
Age	Mean ± SD,	20.4 ± 2.9
	Median, Range	19, 18–37
Gender	Female	115 (71.0)
	Male	46 (28.4)
	Other	1 (0.6)
Race/Ethnicity	Non-Hispanic White	60 (37.0)
	Nonwhite:	102 (63.0)
	African-American/Black	41 (25.3)
	Other (incl. Hispanic)	43 (26.5)
	Asian	11 (6.8)
	Native American	6 (3.7)
	Pacific Islander	1 (0.6)
Class level	Freshman	106 (65.4)
	Sophomore	18 (11.1)
	Junior	12 (7.5)
	Senior	26 (16.0)
Major	Health Sciences	50 (30.9)

As indicated in **Table 2**, participants generally had a very good knowledge of the main modes of COVID-19 transmission and common symptoms of the disease. Close to two-thirds identified correct answers to all knowledge questions in the survey. Additionally, to a great extent students agreed with, and were following social distancing measures currently instituted. The most trusted sources of information were official sources: government (77.8%), followed by medical professionals (58.0%). These were also the most commonly used sources of information about the COVID-19 pandemic. Only 12.3% said they trusted social media as a source of information. Almost universally, students had changed their behaviors in response to the pandemic, including by increasing hand washing and limiting social contacts (100%), and starting to wear masks (97.5%).

**Table 2:** Descriptive statistics for observed indicators of COVID-19 knowledge, sources of information, behaviors, academic and life difficulties, and mental health burden (N = 162).

Variable	Category	N (%)
**I. Knowledge**		
Knowledge statements, correctly answered (True/False)	Correct answers provided to all statements	104 (64.2)
	1. The COVID-19 virus spreads via respiratory droplets of infected individuals (T)	151 (93.2)
	2. The main clinical symptoms of COVID-19 are fever, fatigue, and dry cough (T)	159 (98.1)
	3. There is effective cure for COVID-19 (F)	150 (92.6)
	4. Early symptomatic and supportive treatment can help most patients recover from the infection (T)	145 (89.5)
	5. All people with COVID-19 will develop severe Illness (F)	139 (85.8)
	6. Those who are elderly and have chronic illnesses are more likely to be severe cases (T)	160 (98.8)
	7. Those with COVID-19 cannot transmit the virus to others when a fever is not present (F)	154 (95.1)
Level of agreement with social distancing measures (agree/strongly agree)	1. To prevent infection with COVID-19, people should avoid going to crowded places	159 (98.2)
	2. Isolation of people who are infected with COVID-19 are effective ways to reduce the spread of the virus	151 (93.2)
	3. Young adults should take measures to prevent infection with COVID-19	133 (82.1)
**II. Sources of Information (n = 156)**		
Trust in sources of information	Official sources	141 (87.0)
	Government	126 (77.8)
	Health professionals	94 (58.0)
	Unofficial sources	37 (22.8)
	Social media	20 (12.3)
	Friends and family	32 (19.8)
	News sources	46 (28.4)
Use of sources of information (often/always)	Official sources	123 (75.9)
	Government sites	90 (57.7)
	Health professionals	105 (67.3)
	Unofficial Sources	74 (45.7)
	Social media	48 (31.8)
	Friends and family	51 (32.7)
	News websites	70 (44.8)
Number of hours/day looking for information on COVID-19 (**n = 154**)	On news websites (Mean ± SD, median, IQR)	(1.5 ± 2.1, 1, 1–2)
	On social media (Mean ± SD, median, IQR)	(1.9 ± 3.4, 1, 0–2)
**III. Behaviors** (**n = 158)**		
Changed behaviors, even a little bit	Increased hand washing	158 (100.0)
	Increased spending on cleaning supplies	152 (96.2)
	Limited going out	156 (98.7)
	Stocked up on food and supplies	154 (97.5)
	Started wearing a mask	154 (97.5)
	Limited social outings	158 (100)
	Avoided going to doctor or dentist	148 (93.7)
**IV. Academic and Life Difficulties**		
Academic Difficulties	Ability to focus and complete academic work	126 (77.8)
	Ability to focus on academic work	119 (73.5)
	Completing assignments and tests	83 (51.2)
	Difficulty with online learning/inadequate access	99 (61.1)
	Difficulties with online mode of learning	95 (58.6)
	Inadequate WiFi/Computer access	36 (22.2)
	No academic difficulties	22 (13.6)
Everyday Life Difficulties	Lost job, reduced wages or work hours	92 (56.8)
	Getting food	36 (22.2)
	Obtaining hygiene supplies and medications	96 (59.3)
	Transportation	18 (11.1)
**V. Level of Concern and Mental Health Burden**		
Level of concern with the COVID-19 pandemic	Not at all to moderately	54 (33.3)
	Very to extremely concerned	108 (66.7)
		(Mean ± SD)
Mental health burden (**n = 151**)	BSI Depression	64.4 ± 10.7
	BSI Anxiety	58.2 ± 13.6
	BSI Somatization	53.5 ± 11.9
	Stress	20.6 ± 7.3

Note: BSI–Brief Symptoms Inventory 18, values represent T scores based on gender specific community norms.

A majority of students reported experiencing academic difficulties since the start of the pandemic. Ability to focus on academic work (73.5%) and difficulties with online learning (58.6%) were the most commonly cited issues related to academics. With respect to difficulties in everyday life, obtaining hygiene supplies and medications (59.3%), and losing job/work hours/reduced wages (56.8%) were the most common life difficulties. A number of students (22.2%) also reported difficulties in getting food. Two-thirds of participants (66.7%) were greatly concerned about COVID-19 epidemic. Finally we report levels of depression, anxiety, somatization, and perceived stress reported by the study participants.

#### 3.2. Bivariate Associations with mental health burden variables

Bivariate associations between demographic characteristics, knowledge and concerns, precautionary behaviors, and hardships experienced during the pandemic and mental health burden variables are presented in **Table 3**. With respect to demographic characteristics, non-freshmen (sophomores, juniors, and seniors) were more likely to exhibit increased levels of anxiety compared to freshmen, and female students reported significantly higher levels of stress than their male counterparts. Spending a greater amount of time looking for COVID-19 information on news sites was associated with increased level of anxiety and somatization, while increased level of anxiety was also associated with spending greater hours looking for information on social media. Interestingly, the number of hours spent on news sites looking for information on COVID-19 was significantly correlated with the amount of time spent on social media looking for COVID-19 information (ρ = 0.77, p<0.001). Those who reported trust in the news media were less likely to exhibit increased levels of somatization.

**Table 3:** Bivariate associations of selected socio-demographic variables, knowledge and sources of information, behaviors and academic difficulties with mental health burden among undergraduate college students during COVID-19 epidemic. (N = 151).

		Mental Health Burden (mean ± SD)			
		BSI Depression	BSI Anxiety	BSI Somatization	Stress
Variables	Categories				
**I Demographics**					
Age (continuous)^**†**^		0.01 *ns*	0.10 *ns*	0.06 *ns*	-0.06 *ns*
Gender	Female	64.4 ± 9.9	58.1 ± 12.6	53.6 ± 11.6	21.8 ± 7.0
	Male	64.9 ± 12.4 *ns*	58.7 ± 15.7 *ns*	53.3 ± 12.1 *ns*	18.1 ± 7.5** p = 0.004
Race	White	63.9 ± 11.6	58.3 ± 13.7	53.5 ± 12.2	20.8 ± 8.1
	Non-White	64.8 ± 10.1 *ns*	58.1 ± 13.6 *ns*	53.4 ± 11.8 *ns*	20.5 ± 6.9 *ns*
Class Level	Freshman	64.2 ± 11.1	56.4 ± 13.9	53.3 ± 12.0	20.6 ± 7.3
	Other (sophomore, junior, and senior)	65.0 ± 9.7 *ns*	61.7 ± 12.3* p = 0.024	53.7 ± 11.8 *ns*	20.8 ± 7.5 *ns*
Major	Health	63.5 ± 10.3	57.2 ± 13.3	53.5 ± 10.5	21.8 ± 7.2
	Other	64.8 ± 10.8 *ns*	58.6 ± 13.7 *ns*	53.4 ± 12.5 *ns*	20.1 ± 7.4 *ns*
**II Knowledge**					
Correct answers provided to all knowledge statements	No	63.4 ± 10.6	58.6 ± 14.2	54.4 ± 11.7	19.2 ± 7.9
	Yes	65.0 ± 10.7 *ns*	57.9 ± 13.3 ns	52.9 ± 12.0 ns	21.4 ± 6.9 ns
**III Sources of information**					
Trust official (govern., medical professionals) sources	No	67.9 ± 11.0	60.1 ± 13.6	55.2 ± 12.5	19.9 ± 8.1
	Yes	64.1 ± 10.8 *ns*	58.0 ± 13.6 *ns*	53.2 ± 11.9 *ns*	20.7 ± 7.3 *ns*
Trust news media	No	64.8 ± 11.1	58.6 ± 14.1	54.7 ± 12.5	20.9 ± 7.9
	Yes	63.6 ± 9.7 *ns*	57.2 ± 12.3 *ns*	50.5 ± 9.8* p = 0.046	20.2 ± 6.0 *ns*
Trust unofficial (social media, friends/family) sources	No	63.7 ± 11.0	57.8 ± 13.7	52.8 ± 12.2	20.6 ± 7.2
	Yes	62.6 ± 9.2 *ns*	59.2 ± 13.4 *ns*	55.5 ± 10.8 *ns*	20.7 ± 7.8 *ns*
Use official (govern., medical professionals) sources	No	62.3 ± 12.0	54.9 ± 13.8	52.1 ± 12.1	18.7 ± 8.1
	Yes	65.0 ± 10.3 *ns*	59.0 ± 13.5 *ns*	53.8 ± 11.9 *ns*	21.2 ± 7.1 *ns*
Use news media	No	65.3 ± 10.8	58.4 ± 14.0	52.7 ± 12.3	20.8 ± 7.2
	Yes	63.4 ± 10.5 *ns*	58.0 ± 13.2 *ns*	54.4 ± 11.5 *ns*	20.5 ± 7.6 *ns*
Use unofficial (social media, friends/family) sources	No	64.8 ± 11.2	58.3 ± 14.3	53.4 ± 12.2	21.0 ± 7.0
	Yes	64.0 ± 10.0 *ns*	58.1 ± 12.9 *ns*	53.5 ± 11.7 *ns*	20.3 ± 7.7 *ns*
Time spent on looking for information on news sites	≤1 hour	63.7 ± 11.3	55.9 ± 13.9	52.2 ± 12.1	20.7 ± 7.2
	>1 hour	66.2 ± 9.0 *ns*	63.5 ± 11.3***	56.4 ± 10.9*	20.5 ± 7.7 ns
			p = 0.001	p = 0.049	
Time spent on looking for information on social media	≤1 hour	63.4 ± 11.0	56.6 ± 13.8	52.6 ± 12.0	20.5 ± 7.1
	>1 hour	66.7 ± 9.6 ns	61.7 ± 12.6*	55.3 ± 11.5 ns	21.1 ± 8.0 ns
			p = 0.032		
**IV Academic and Life difficulties**					
Academic difficulties—Ability to focus on academic work	No	56.3 ± 10.8	50.4 ± 11.6	47.5 ± 9.7	16.2 ± 6.2
	Yes	66.4 ± 9.7*** p < 0.001	60.0 ± 13.4*** p < 0.001	54.9 ± 12.0** p = 0.003	21.7 ± 7.2*** p < 0.001
Academic difficulties—Difficulties with online learning	No	59.9 ± 11.0	54.5 ± 13.1	50.9 ± 10.9	18.6 ± 8.1
	Yes	67.1 ± 9.5*** p < 0.001	60.3 ± 13.5** p = 0.011	54.9 ± 12.3* p = 0.047	21.9 ± 6.6** p = 0.007
Everyday difficulties—Lost job, reduced wages or work hours	No	60.2 ± 10.6	55.1 ± 12.8	52.6 ± 9.3	19.5 ± 6.6
	Yes	67.4 ± 9.7*** p < 0.001	60.3 ± 13.8* p = 0.019	54.1 ± 13.4 *ns*	21.4 ± 7.7 *ns*
Everyday difficulties–Obtaining medications and hygiene supplies	No	62.3 ± 11.6	55.1 ± 14.1	51.3 ± 12.0	18.3 ± 8.0
	Yes	65.8 ± 9.8* p = 0.049	60.1 ± 12.9* p = 0.027	54.8 ± 11.7 *ns*	22.1 ± 6.5** p = 0.002
Everyday difficulties–Obtaining food	No	63.5 ± 11.1	57.3 ± 13.9	53.0 ± 12.1	20.3 ± 7.6
	Yes	67.3 ± 8.5 *ns*	61.1 ± 12.3 *ns*	54.8 ± 11.2 *ns*	21.9 ± 6.2 *ns*
**V Level of concern**					
Concerned about COVID-19	Not at all to moderately	62.5 ± 11.8	55.1 ± 13.4	49.5 ± 10.5	19.4 ± 8.0
	Very to extremely	65.4 ± 10.0 *ns*	59.7 ± 13.5* p = 0.05	55.4 ± 12.1** p = 0.004	21.3 ± 7.0 *ns*

Note

^**†**^ Pearson correlation coefficient; *ns* = non-significant

* p < 0.05

** p < 0.01

*** p < 0.001.

Academic difficulties (the ability to focus on academic work and online learning) were associated with the increased levels of all four mental health burden measures. Those with increased levels of depression and anxiety were also more likely to report having lost job, wages, or work hours, and difficulties in obtaining medications and hygiene supplies. Increased levels of stress were also associated with difficulties in obtaining medications and hygiene supplies. Finally, those participants who expressed higher levels of concern about COVID-19 were more likely to report higher levels of anxiety and somatization.

### Multivariable associations

**Table 4** presents results from the linear regression models for each of mental health burden outcomes. Analyses indicated that economic hardship was the most significant predictor of depression among respondents, followed by difficulties with focusing on academics (see Table 4 for test statistics). Anxiety levels were significantly higher among non-freshmen, those who spent more than one hour per day looking for COVID-19 information on news sites, and ability to focus on academic work. Ability to focus on academic work was also a significant predictor of somatic problems, together with being very to extremely concerned about COVID-19. Interestingly, those who exhibited trust in news media were less likely to exhibit somatic problems. Finally, male sex was significantly associated with lower levels of stress, while inability to focus on academic work, and difficulties with obtaining medicine and hygiene supplies were significant predictors of higher levels of stress among undergraduate students.

**Table 4:** Multiple linear regression analyses of predictors of mental health burden among undergraduate college students during COVID-19 epidemic.

	BSI-Depression		BSI-Anxiety		BSI-Somatization		Stress	
Variable	β	(95% CI)	β	(95% CI)	β	(95% CI)	β	(95% CI)
Gender (Male = 1)	-	-	-	-	-	-	-0.17	(-0.32- -0.01)* p = 0.036
Class Level (> freshmen = 1)	-	-	0.16	(0.01–0.31)* p = 0.039	-	-	-	-
Trust news media (Yes = 1)	-	-	-	-	-0.17	(-0.32- -0.02)* p = 0.024	-	-
Time spent looking for information on news sites (> 1hr = 1)	-	-	0.21	(0.04–0.38)* p = 0.016	0.12	(-0.03–0.27) *ns*	-	-
Time spent looking for information on social media (> 1hr = 1)	-	-	0.03	(-0.14–0.2) *ns*	-	-	-	-
Academic difficulties—Ability to focus on academic work (Yes = 1)	0.24	(0.09–0.42)** p = 0.003	0.17	(0.00–0.35)* p = 0.047	0.21	(0.04–0.39)* p = 0.014	0.18	(0.02–0.37)* p = 0.033
Academic difficulties—Difficulties with online learning (Yes = 1)	0.16	(-0.04–0.32) ns	0.10	(-0.06–0.27) *ns*	0.08	(-0.09–0.24) *ns*	0.12	(-0.04–0.29) *ns*
Everyday difficulties—Lost job, reduced wages or work hours (Yes = 1)	0.25	(0.1–0.40)*** p = 0.001	0.12	(-0.04–0.28) *ns*	-	-	-	-
Everyday difficulties–Obtaining medications and hygiene supplies (Yes = 1)	0.13	(-0.02–0.28) *ns*	0.13	(-0.02–0.29) *ns*	-	-	0.18	(0.02–0.33)* p = 0.024
Everyday difficulties–Obtaining food (Yes = 1)	-	-	*-*	-	-	-	-	-
Concerned about COVID-19 (Very to extremely)	-	-	0.08	(-0.07–0.23) *ns*	0.21	(0.05–0.36)** p = 0.008	-	-
**Test Statistics**	F(4,146) = 11.75; p<0.001; R^2^ = 0.24		F(8,142) = 4.87; p<0.001; R^2^ = 0.22		F(5,145) = 5.50; p<0.001; R^2^ = 0.16		F(4,145) = 6.98; p<0.001; R^2^ = 0.16	

Note: All dichotomous variables were coded 0 or 1. *ns–*not significant

* p < 0.05

** p < 0.01

*** p < 0.001.

## Discussion

Previous studies have shown that public health emergencies have a significant impact on mental health of college students [28, 35]. Since the COVID-19 outbreak, a few studies have emerged describing higher levels of anxiety and increased risk perception among college students during COVID-19 pandemic [26, 27]. The current study is among the first to examine the impact of the COVID-19 pandemic on mental health among undergraduate college students in the U.S. Northern New Jersey has been one of the most severely affected regions in the U.S. by the current pandemic, creating uncertainty, anxiety and stress among a wider population. The pandemic has also elevated concerns about well-being of the members of the university community, including students. While previous studies have indicated that the current pandemic may have widespread impacts on students’ learning experiences [8, 36], our results indicate that college students who are experiencing considerable number of academic and everyday difficulties during the COVID-19 pandemic also report increased levels of mental health burden. This is of potential concern as the pandemic is occurring against the backdrop of increasing mental health issues among college students [37]. Additional stress may lead to further detrimental effects on the learning experiences and mental health of undergraduates.

Most of our participants were females, which is not surprising considering that the student body at the university is approximately 60% female. As this was an introductory personal health course, the majority of participants were freshmen. The racial composition of the sample was also reflective of the University’s diverse undergraduate student body. In multivariable models, students other than freshmen were more likely to report higher levels of anxiety. A possible explanation for this could be that students in upper classes may be more concerned about the impact of the pandemic on their post-graduation plans and the economy. Additionally, male students were less likely to report higher levels of stress. This is similar to previous studies’ findings that, in general, female students report higher perceived stress levels than their male counterparts [38, 39]. Traditional self-concepts of masculinity and femininity can lead to differential expression of attitudes and emotions towards life experiences [40]. Therefore, it is possible that in circumstances surrounding COVID-19 pandemic female students were more likely to express internalized disorders such as stress.

Change of behavior in response to the pandemic was pervasive. In accordance with the current recommendations, our respondents almost universally increased hand washing, limited social outings, and started wearing masks. This is contrary to the findings of a recent study of adults in the U.S. with chronic conditions [41], but similar to behavior changes observed in two population studies in Hong Kong [42] and China [43]. While some of the changes in behavior may be the result of mandatory requirements (i.e. lockdowns, mandatory mask wearing), it is also possible that students taking a personal health course are more conscious of the way virus spreads and are more likely to adapt to behaviors that would prevent the spread of the virus.

While we found no significant association of knowledge levels with mental health burden variables, the descriptive findings of this study indicate that undergraduate students possessed an adequate level of knowledge related to COVID-19 at the time of the survey administration. Nearly all respondents correctly identified how the virus spreads, clinical symptoms of the infection, or the most vulnerable populations to an infection. Information surrounding COVID-19 pandemic has been characterized as an “infodemic” [44] with an unprecedented amount of information but also misinformation, particularly present on social media. Yet, participants in this study were more likely to report use of “formal” sources of information as opposed to “informal” or news sites, resulting in higher correct knowledge of COVID-19. Additionally, students who are enrolled in personal health course may be more likely to be attuned to correct information related to COVID-19. The most common misconception was that those with COVID-19 cannot transmit the virus to others when a fever is not present (35.8% of participants endorsed this statement). Another misconception exhibited by a smaller number (18.5%) of participants was that young people do not necessarily need to take measures to prevent infection with COVID-19. Educating students on the role that asymptomatic and pre-symptomatic individuals play in transmission of COVID-19 should be a priority for college health educators, as returning to campus may necessitate limits on students’ gathering in confined settings, or institute mask-wearing to mitigate spread of the virus.

Overall, official sources, such as health professionals (64.8%) and government sites (55.6%), were the most common sources of information regarding COVID-19 among undergraduate students. Furthermore, official sites were the most trusted sources of information (87.0%). Similar to the study of adult U.S. population [45], social media was the least trusted source of COVID-19 information in this sample (12.3%), which is encouraging as social media platforms may have a high potential for spreading misinformation [44]. It is also possible that users searching for information on social media are directed to reliable sources such as the CDC [46]. In multivariable regression models, spending a greater amount of time looking for COVID-19 information on the news sites was associated higher levels of anxiety. This is consistent with previous studies which have found that increased time spent using social media per day was associated with increased odds of reporting high levels of anxiety [47]. While we did not test for possible exposure to COVID-19 virus, it is possible that those who perceived as being exposed to virus were more likely to search for information. It is also possible that highly anxious individuals may engage in more information-seeking as an adaptive coping strategy [48]. However, trusting news media was associated with decreased levels of perceptions of bodily dysfunctions. News sites presenting substantiated and accurate COVID-related information may have the potential to create a positive environment that is conducive to easing somatic distress in response to psychosocial stress.

Academic difficulties, such as ability to focus on academic work, were significantly associated with increased levels of depression, anxiety, somatization, and stress. It is also important to note that, at the bivariate level, all four forms of mental health burden were significantly associated with online learning difficulties. However, once the effects of other covariates were considered, difficulty with online learning was no longer a significant predictor of mental health burden. Higher level of depression were also significantly associated with loss of job, reduced wages or work hours, while difficulties in obtaining medicine and hygiene supplies were significantly associated with increased stress levels. Students who are dependent on jobs to support themselves and/or families may be particularly vulnerable to depression and worry due to economic hardship. As uncertainties about the future continue this may lead to worsening mental health status, particularly among young individuals [49]. The current pandemic has shifted students’ priorities. Many are worried about their own health, health of their families, or struggling financially, perhaps making them less focused on academics, and increasing academic difficulties. An abrupt migration of traditional face-to-face courses to the on-line mode may be particularly difficult for students who are used to in person classes and to those from academically marginal undergraduate student groups [50], or in courses that are poorly suited for online learning [51]. Struggling academically with online courses may further exacerbate mental health distress among students. All of this may have implications for students’ retention rate, leaving college administrators and instructors with the task to create innovative solutions to the unexpected challenge the pandemic has created. College support services may have a crucial role in helping students navigate the life challenges associated with the pandemic may help to improve their mental health.

### Limitations

This study has several limitations worth noting. The study design is cross-sectional. Therefore, the causality cannot be established, as data represents a single moment in time. Given the limited resources available and time-sensitivity of the COVID-19 outbreak, the sample is comprised of undergraduates who are enrolled in one introductory core curriculum course. While the course is university-wide, our results may not generalize to other students. Participation in the study may have been influenced by various difficulties students found themselves in. The data are based on self-report and may be subject to social desirability bias. Our questions captured only fundamental knowledge, attitudes, and a limited set of behaviors. It is possible that our questions tapped into personal beliefs and/or endorsement of health promotion guidelines by the authorities. Future studies should also capture whether direct or indirect exposure to COVID-19 virus added to mental health distress. Additionally, self-reported levels of mental health distress are not confirmed by the mental health professionals’ assessments.

### Conclusions

Our findings support the notion that the current COVID-19 pandemic is making a significant negative impact on mental health of college students. College students who exhibit greater academic and life difficulties may be particularly vulnerable to higher mental health distress. The current pandemic may further exacerbate already existing problems. The timeline of the pandemic is uncertain further impacting students’ academics, lives, and mental health. With a host of negative consequences associated with poor mental health, further research is needed to address additional risk factors (i.e., substance use, coping mechanisms, social support, family and peer relationships dynamics) that are associated with mental health in this population. Additional studies investigating the effect of pandemic on mental health of faculty may provide a better understanding of the impact of COVID-19 on higher education. Our findings suggest that college health service providers and administrators need to consider proactive measures to support the mental health and well-being of students. Mental health interventions and professionally trained counselors could help students address academic and financial concerns, which may alleviate mental health burden of the COVID-19 pandemic. In public health emergencies like this, many students will have special needs and emerging challenges that will require responsive programming by colleges.

## Supporting information

S1 File (DOCX)

S2 File (XLS)
