# Supplementary material for: The impact of the COVID-19 epidemic on mental health of undergraduate students in New Jersey, cross-sectional study
Source: PLoS One. 2020 Sep 30;15(9):e0239696. doi: 10.1371/journal.pone.0239696 (PMC7526896; doi:10.1371/journal.pone.0239696)
Supplement: S1 File — (DOCX) [file pone.0239696.s001.docx]

**Project Title: The impact of COVID-19 epidemic on college students**

**Principal Investigator: Dr. Alex Kecojevic**

**Investigator e-mail: kecojevica@wpunj.edu**

**Department: Public Health**

**Protocol Approval Date:**

**IRB Contact Phone #: 973-720-2852**

**Consent Statement:**

You are invited to participate in a survey conducted by the researchers from Public Health Department at WPUNJ. You will be presented with questions on the current coronavirus outbreak.  In this survey we hope to learn how has COVID-19 impacted college students. Your participation in this survey is completely voluntary and confidential. No personal information will be collected or reported so feel free to answer honestly. The objective of this research is to inform future public health efforts, particularly public health campaigns for the college students.

Risks and Benefits: A potential risk of your participation in the study is that you may feel uncomfortable answering some questions. However, all of information that you provide is confidential. There are no direct benefits to you from participating in this study. However, the information you provide may help improve public health efforts with regards to the new coronavirus and, thus, reduce harm from this epidemic.

Payments: At the end of current survey, you will be asked whether you would like to enter in a drawing for one of five $20 gift cards that will be e-mailed to you. You are asked to provide your e-mail only (preferably non-WPUNJ e-mail). Your e-mail is not linked in any way to your responses in this survey. By participating in this survey you accept above risks and benefits.

Participants Rights: If you have read this form and have decided to participate in this project, please understand your participation is voluntary and you have the right to withdraw your consent or discontinue participation at any time without penalty or loss of benefits to which you are otherwise entitled. You understand that any data collected as part of this study will be stored in a safe and secure location, and that this data will be destroyed when this research is completed. The alternative is not to participate. You have the right to refuse to answer particular questions. The results of this research study may be presented at scientific or professional meetings or published in scientific journals. Your individual privacy will be maintained in all published and written data resulting from the study. You may e-mail the study's investigator Dr. Alex Kecojevic to discuss any questions you may have regarding this survey.

**Consent:**If you are 18 years of age or older, understand the statements above, and will consent to participate in the study, click on the "I Consent" button to begin the questionnaire. If you do not wish to participate in this study, please click on the "I Do Not Consent" button to exit this survey.

- **I Consent**
- **I Do Not Consent**

**I Demographics:**

**Q1: Please tell us your sex:**

- Male
- Female
- Other
- Prefer Not to Answer

**Q2: How old are you? (in years)**

- ENTER AGE

**Q3 Are you a:**

- Freshman
- Sophomore
- Junior
- Senior
- Graduate
- Not Applicable (Employee)

**Q4 Are you Spanish, Hispanic, or Latino?**

- Yes
- No

**Q5 What is your race?**

- African-American or Black
- American Indian or Alaska Native
- Asian
- Native Hawaiian or Pacific Islander
- White/Caucasian
- Multiracial
- Other

**Q6. Is your major related to science or health?**

- Yes
- No

**II Knowledge (True/False) (or level of agreement):**

Q1. The COVID-19 virus spreads via respiratory droplets of infected individuals (T)

Q2: The main clinical symptoms of COVID-19 are fever, fatigue, and dry cough (T)

Q3. There is effective cure for COVID-19 (F)

Q4. Early symptomatic and supportive treatment can help most patients recover from the infection (T)

Q5. All persons with COVID-19 will develop severe cases. (F)

Q6. Those who are elderly and have chronic illnesses are more likely to be severe cases (T)

Q7. Persons with COVID-19 cannot transmit the virus to others when a fever is not present (F)

Q8-Q10: Strongly Agree to Strongly Disagree scale
Q8. It is not necessary for young adults to take measures to prevent infection with COVID-19

Q9. To prevent infection with COVID-19, people should avoid going to crowded places and avoid public transportation

Q10. Isolation of people who are infected with COVID-19 are effective ways to reduce the spread of the virus

**III Behaviors (level of agreement):**

**Since the start of epidemic:**

Q1. I have increased hand washing.

Q2. I increased spending on cleaning supplies

Q3. I have limited going outside only to essential trips (i.e. grocery)

Q4. I stocked up on food and supplies

Q5. If I need to go out I always wear mask

Q6. I limited social contacts with my friends

Q7. I avoid going to the doctor or dentist for routine appointments

**IV Experience**

**Q1. Have you experienced any academic difficulties due to the coronavirus crisis? (Select all that apply):**

- Ability to focus on academic work
- Difficulties with online learning
- Inadequate WiFi/Computer access
- Completing assignments and tests
- Communication with instructors
- Other: ____________________

**Q2. Have you experienced any life difficulties due to the coronavirus crisis? (Select all that apply):**

- Reduced wages or work hours
- I have lost my job
- Childcare
- Getting food
- Getting hand sanitizer or cleaning supplies
- Getting routine/essential medications
- Transportation
- Other: __________________

**Q3. What sources do you trust to provide accurate COVID-19 information? (Select all that apply):**

- Official government websites (i.e. NJ Department of Health, CDC, etc.)
- County health department
- Social media (Facebook, Twitter, Instagram)
- News websites
- Friends or family members
- Doctors
- Other

**Q4.** **How often do you use each of the following sources to get health information regarding COVID-19 epidemic? (Never, Rarely, Sometimes, Often, Always)**

1. Family and/or friends

2. Health or medical professional

3. Social Media

4. News media

5. Government web sites

**Q5. How many hours a day do you spend searching the Internet for news regarding COVID-19?** _____ (hours)

**Q6. How many hours a day do you spend searching looking at social media for news regarding COVID-19?**_____ (hours)

**Q7. How concerned do you feel about the novel coronavirus, COVID-19?**

- Not at all concerned
- A little concerned
- Moderately concerned
- Very concerned
- Extremely concerned

**V. Psychological Impact (Anxiety, Depression and Stress).**

**A. Anxiety and Depression BSI Scale**

Scale: 1. Not at all; 2. A little bit; 3. Moderately; 4. Quite a bit; 5. Extremely

**6. Refuse to answer**

This is a list of problems that people may experience during these times. Please indicate how much this problem has distressed or bothered you during the 7 days, including today. During the past 7 days, how much were you distressed by:

1 Faintness or dizziness

2 Feeling no interest in things

3 Nervousness or shakiness inside

4 Pains in the heart or chest

5 Feeling lonely

6 Feeling tense or keyed up

7 Nausea or upset stomach

8 Feeling blue

9 Suddenly scared for no reason

10 Trouble getting your breath

11 Feelings of worthlessness

12 Spells of terror or panic

13 Numbness or tingling in parts of your body

14 Feeling hopeless about the future

15 Feeling so restless you couldn't sit still

16 Feeling weak in parts of your body

17 Thoughts of harming yourself

18 Feeling fearful

**B. STRESS Scale**

Answer scale: 1. Never; 2. Almost never; 3. Sometimes; 4. Fairly often; 5. Very often

88. Refuse to answer

The questions in this scale ask you about your feelings and thoughts since COVID-19 epidemic started. In each case, please indicate how often you felt or thought a certain way. Since the COVID-19 epidemic started...

1. How often have you been upset because of something that happened unexpectedly?

How often have you felt that you were unable to control the important things in your life?

How often have you felt nervous and "stressed"?

How often have you felt confident about your ability to handle your personal problems?

How often have you felt that things were going your way?

How often have you found that you could not cope with all the things that you had to do?

How often have you been able to control irritations in your life?

How often have you felt that you were on top of things?

How often have you been angered because of things that were outside of your control?

How often have you felt difficulties were piling up so high that you could not overcome them?
